# Impact of splenic artery ligation after major hepatectomy on liver function, regeneration and viability

**DOI:** 10.1038/srep34731

**Published:** 2016-10-11

**Authors:** Jorge Carrapita, Ana Margarida Abrantes, Sofia Campelos, Ana Cristina Gonçalves, Dulce Cardoso, Ana Bela Sarmento-Ribeiro, Clara Rocha, Jorge Nunes Santos, Maria Filomena Botelho, José Guilherme Tralhão, Olivier Farges, Jorge Maciel Barbosa

**Affiliations:** 1 General Surgery Department of Vila Nova de Gaia/Espinho Hospital, Portugal; 2 Institute of Biomedical Sciences Abel Salazar, University of Oporto, Portugal; 3 Biophysics Institute, Faculty of Medicine, University of Coimbra, Portugal; 4 CNC.IBILI, Faculty of Medicine, University of Coimbra, Portugal; 5 Centre of Investigation on Environment, Genetics and Oncobiology (CIMAGO), Portugal; 6 Pathologic Anatomy Department of Vila Nova de Gaia/Espinho Hospital, Portugal; 7 Laboratory of Oncobiology and Hematology (LOH), University Clinic of Hematology and Appied Molecular Biology Unit, Faculty of Medicine, University of Coimbra, Portugal; 8 Nuclear Medicine Department, University Hospital of Coimbra, Portugal; 9 Clinical Hematology Department, Coimbra University Hospital Centre (CHUC), Portugal; 10 ESTESC-Coimbra Health School Department Complementary Sciences, Polytechnic Institute of Coimbra, Portugal; 11 Institute for Systems Engineering and Computers at Coimbra (INESCC), Portugal; 12 Surgery A, Surgery Department of Coimbra University Hospital, Faculty of Medicine, University of Coimbra, Portugal; 13 Hepatobiliary and pancreatic unit, Beaujon Hospital, AP-HP, Université Paris Clichy, France; 14 Fernando Pessoa University, Oporto, Portugal

## Abstract

It was reported that prevention of acute portal overpressure in small-for-size livers by inflow modulation results in a better postoperative outcome. The aim is to investigate the impact of portal blood flow reduction by splenic artery ligation after major hepatectomy in a murine model. Forty-eight rats were subjected to an 85% hepatectomy or 85% hepatectomy and splenic artery ligation. Both groups were evaluated at 24, 48, 72 and 120 post-operative hours: liver function, regeneration and viability. All methods and experiments were carried out in accordance with Coimbra University guidelines. Splenic artery ligation produces viability increase after 24 h, induces a relative decrease in oxidative stress during the first 48 hours, allows antioxidant capacity increment after 24 h, which is reflected in a decrease of half-time normalized liver curve at 48 h and at 72 h and in an increase of mitotic index between 48 h and 72 h. Splenic artery ligation combined with 85% hepatectomy in a murine model, allows portal inflow modulation, promoting an increase in hepatocellular viability and regeneration, without impairing the function, probably by inducing a less marked elevation of oxidative stress at first 48 hours.

Major hepatectomy (MH), traditionally defined as the resection of 3 or more segments, is currently the standard treatment for liver benign or malignant diseases, allowing also collecting grafts in living donor liver transplantation (LDLT)^1^.

Several factors have been implicated in the increasing survival rate of patients undergoing major hepatectomy, as it has been shown in the last 15 years^1^,^2^,^3^. Examples of this are: implementation of multidisciplinary oncosurgical strategies, greater efficacy of chemotherapeutic drugs, whole range of instrumentation and techniques used in perioperative period, better accuracy in selection of candidates for hepatectomy/transplantation through a rigorous evaluation of preoperative liver function^4^,^5^,^6^ and more precise estimation of future liver remnant (FLR), besides its increment through selective portal embolization or iterative hepatectomy^7^,^8^.

Despite technical advancement, posthepatectomy mortality remains a concern, being the main cause of liver failure^1^,^7^. In fact, the greatest limitation of liver neoplasms therapeutic strategy is currently the percentage of remaining functional liver after hepatectomy. In other words, the pivotal point of recovery after MH is liver regeneration^2^.

Several reports suggest that postoperative liver failure is due to reduced liver volume and posthepatectomy hemodynamic changes, that induce production of reactive oxygen species (ROS) and inflammatory cytokines, leading to hepatic necrosis and apoptosis^1^,^2^,^9^,^10^. Although adequate portal venous flow and pressure may play a very important role in liver regeneration, these rapid hemodynamic changes can lead to excessive portal flow to residual liver, which may cause high portal pressure, and consequent sinusoidal injury, hepatic parenchymal damages and residual liver hypertrophy accompanied by liver dysfunction^2^,^10^. Similar development has been detected in adult LDLT, presumably due to size mismatch between graft and recipient^9^.

With the increasing of LDLT practice and resection of larger tumours, the “small-for-size” syndrome (SFSS) has emerged as an important clinical problem^2^,^9^,^10^. More recently, it has been proposed the concept of “small-for-flow” based on the assumption that posthepatectomy liver failure (PHLF) and SFSS in LDLT would have the same origin, related to excessive portal flow for residual hepatic parenchyma, generating a sinusoidal overpressure, capillary denudation and bleeding^9^.

Different studies have shown the importance of portal venous pressure (PVP) in the development of liver failure after MH or LDLT, in patients with previous liver damage^10^,^11^,^12^ or in non-cirrhotic livers^1^, focusing the importance of PVP early evaluation after hepatectomy as an independent predictor of liver failure developing after MH^9^,^13^. Considering the pathophysiology of endothelial injury generated by portal overpressure, several strategies have been proposed to modulate portal inflow. In this context, pharmacological trials and surgical techniques have been tested, not only individually but as a complement, namely somatostatin and analogues^14^,^15^,^16^, prostaglandin E_1_^17^,^18^, FK 409 and FTY 720^19^,^20^, terlipressin^21^, inhalation of nitric oxide, carbon monoxide and hydrogen^22^,^23^,^24^, portal caval shunt^25^,^26^ or other portal derivative manoeuvres to prevent its excessive shunting and frequent complications^27^,^28^, plasmapheresis and other plasma purification procedures in order to decrease serum toxin levels^29^,^30^.

However, none of these strategies resulted in a significant survival increase because they have shown an inhibitory effect on liver regeneration after hepatectomy, which limits their use in hepatic surgery^31^. Simultaneously, they have revealed, in animal models, significant morbidity and mortality or they simply do not act in the pathogenic mechanism responsible for these syndromes – portal venous overpressure^29^.

Assuming the splenic blood flow fraction up to 52% of the total portal inflow, its contribution to portal overpressure is evident^32^. Therefore, effects of splenectomy after major hepatectomy have also been investigated^32^,^33^. However, the immunological effect of splenectomy and the incidence of septic complications are still controversial^34^,^35^,^36^. In order to induce less iatrogeny and reduce complications due to splenectomy, tests to evaluate the effects of portal inflow prophylactic modulation through the splenic artery ligation were performed^37^,^38^. These results have been promising, despite confining themselves to case reports or small cohort studies. In a more experimental manner, Ito *et al*. and Irie *et al*. studied the effects of splenic artery ligation in rats submitted to major hepatectomy^39^,^40^. However, additionally to splenic artery ligation, both included a procedure that induces ischemic/reperfusion injury, which is a bias. Furthermore, the first work confines itself to study the injurious effects on liver parenchyma, cell regeneration and survival, while the second one specifically tested the protective effect of the splenic artery ligation through induction of heme oxygenase-1 (HO-1) expression.

For these reasons we propose to perform a wider research on the impact of splenic artery ligation, as a technically feasible manoeuver to modulate the portal inflow after major hepatectomy in a murine model, assessing their effects on hepatocellular function, regeneration and viability. We chose this model because it is the animal model which is the long used concerning hepatocellular regeneration research^41^, of easy manipulation and whose size allows performing radioisotopic evaluations. Additionally, it is a model in which we have great experience.

## Results

### Mortality

The results demonstrated that there was no mortality in a control group of 10 animals subjected to 85% hepatectomy (Hx) with (5 rats) or without ligation of the splenic artery (Hx + Asp) (5 rats), on the seventh postoperative day.

### Hepatic extraction fraction (HEF), time of maximum liver activity (Tmax) and half-time of normalized liver curve (T_1/2_) evaluation by radioisotopic methods

The hepatic extraction fraction (HEF) evaluation after ^99m^Tc-mebofrenin injection allows the study of hepatocyte radiopharmaceutical uptake and excretion kinetics. Therefore, after image processing are obtained different parameters: the time of maximum liver activity uptake (Tmax), the half-time of the normalized liver elimination curve (T_1/2_) and the hepatic extraction fraction (HEF). All these data are able to give accurate information about the dynamic function of the hepatocytes after radiopharmaceutical uptake^5^.

**Figure 1:**
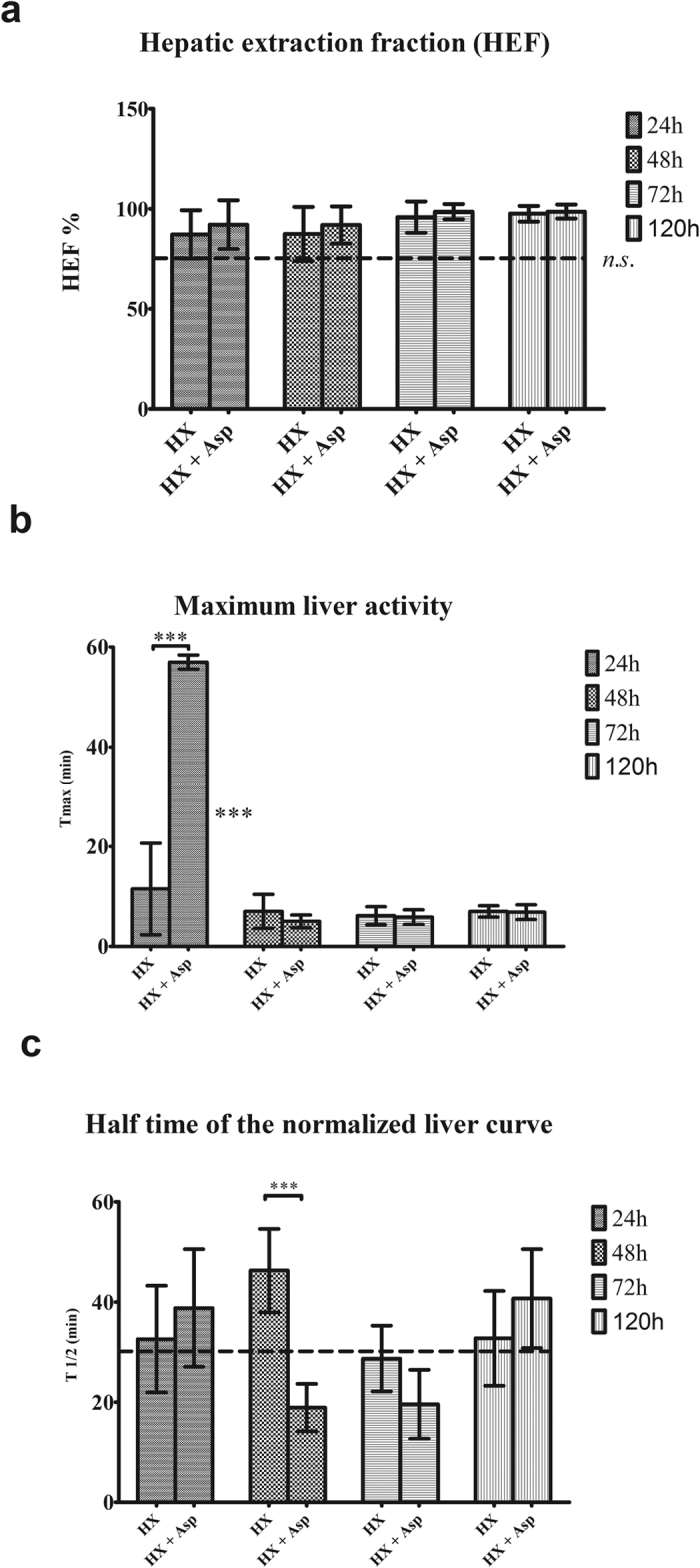
Radioisotopic results. **(a)** Hepatic extraction fraction (HEF) evaluation by radioisotopic methods. (**b)** Time of maximum liver activity (Tmax). **(c)** Half-time of the normalized liver curve. HEF results are expressed as average of percentage and the others are expressed in average of minutes ± standard deviation, from six rats in each group. ***p < 0.001; **p < 0.01; *p < 0.05.

When the HEF was evaluated, there were no differences over time, as shown in Fig. 1, when an 85% hepatectomy was performed. Identical results were obtained when splenic artery ligation was carried out (Fig. 1a). This surgical manoeuver induced an increase on Tmax at first 24 h, which became irrelevant in subsequent times (p < 0.001). However there were no differences between Hx and Hx + Asp groups at 48 hours (Fig. 1b). In addition, for both groups, splenic artery ligation induced a decrease of T_1/2_ at 48 h (p < 0.001) and at 72 h (p = 0.052), with a recovery of this condition at 120 h (Fig. 1c).

### Cell viability

**Figure 2:**
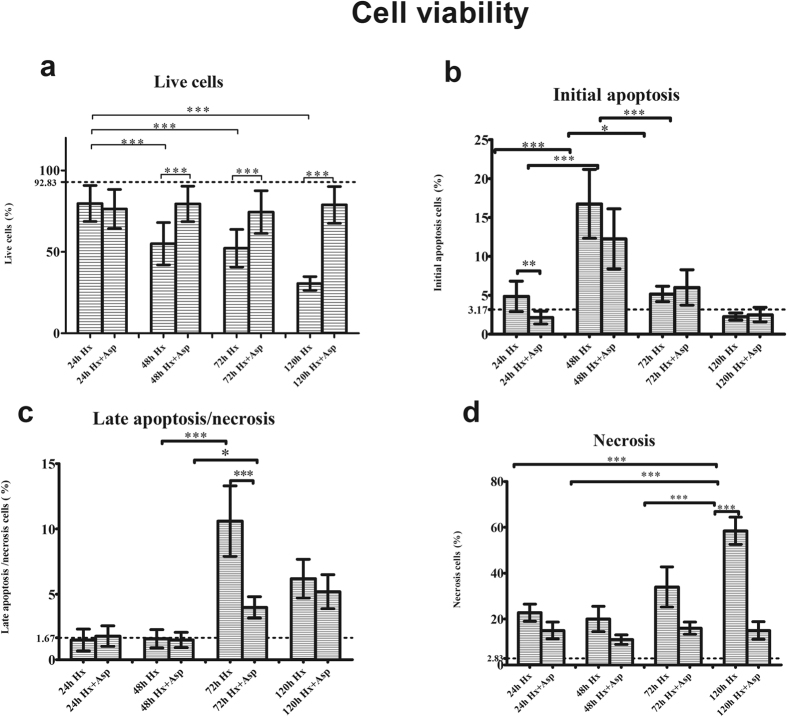
Evaluation of cellular viability and death by flow cytometry. **(a)** Live cells. **(b)** Initial apoptosis. **(c)** Late apoptosis/necrosis. **(d)** Necrosis. Hepatocytes were extracted at 24, 48, 72 and 120 postoperative hours, from rats submitted to hepatectomy (Hx) or hepatectomy + splenic artery ligation (Hx + Asp). Cell death was detected by annexin V and propidium iodide staining and analysed by flow cytometry. Results are expressed as average of percentage of each type of cells ± standard deviation obtained from six rats in each group. ***p < 0.001; **p < 0.01; *p < 0.05. — Baseline value in a control group without any procedure.

In order to evaluate the impact of Hx and Hx + Asp in remnant liver cells, cell viability was assessed by flow cytometry through annexin V/propidium iodide double staining (Fig. 2). As shown in Fig. 2a, there were no significant differences on living cells over time, among Hx + Asp sub-groups. However, when we compared the Hx sub-groups, there was a significant gradual decrease (p < 0.001) over time.

In both groups, there was an increase of initial apoptotic cells (Fig. 2b) at 48 hours (p < 0.001) which decreased over time. However, these changes were more pronounced in sub-groups submitted to splenic artery ligation (p < 0.01 for 48 h–72 h Hx group versus p < 0.001 for 48 h–72 h Hx + Asp group).

As observed in Fig. 2c, at 72 h there was an increase of late apoptosis/necrosis cells in both groups, most prominent in group not submitted to splenic artery ligation (p < 0.001 for Hx group versus p = 0.004 for Hx + Asp group). At 120 h this trend keeps away without statistically significant differences between the groups.

In relation to necrotic cells, there was an increase at 120 h on the Hx group compared with previous times (p < 0.001). On the Hx + Asp group no differences were noticed over time (Fig. 2d).

Comparing Hx with Hx + Asp groups there was a progressive increase of living cells in the group submitted to splenic artery ligation (p < 0.001) (Fig. 2a), an increase of initial apoptosis cells at 24 h more marked on Hx group (p < 0.01) (Fig. 2b), and also an increase of late apoptosis/necrosis cells at 72 h (p < 0.001) (Fig. 2c). It was observed a gradual increase of necrotic cells on the Hx group over time, which became more expressive at 120 h (p < 0.001) (Fig. 2d).

### Oxidative Stress

**Figure 3:**
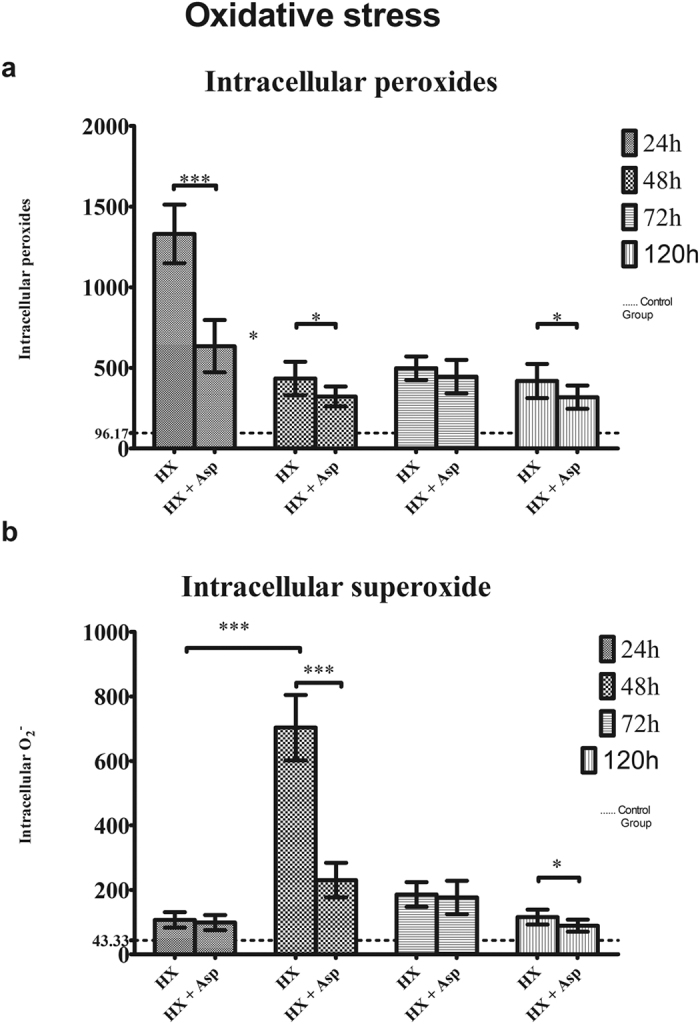
Oxidative stress evaluation. **(a)** Intracellular peroxides. **(b)** Intracellular superoxide. The peroxide and superoxide levels were analysed by flow cytometry using the DCFH2-DA and dihydroethidium (DHE) fluorescent probes. Hepatocytes were extracted at 24, 48, 72 and 120 postoperative hours, from rats submitted to hepatectomy (Hx) or hepatectomy + splenic artery ligation (Hx + Asp). Results are expressed as MFI values ± standard deviation in hepatocytes extracted from six rats in each group. ***p < 0.001; **p < 0.01; *p < 0.05. — Baseline value in a control group without any procedure.

As mentioned before, postoperative liver failure may be related with ROS production. In this context, we analysed intracellular levels of ROS, peroxides and superoxide anion, and GSH (a non-enzymatic antioxidant defence). When we analysed the ROS accumulation (Fig. 3a), at 24 h there was a significant increase of intracellular peroxide production in both groups (p < 0.05), more expressive in the group without splenic artery ligation (p < 0.001). This difference between the two groups remained significant (p < 0.05) at 48 h and at 120 h.

Concerning the results obtained for intracellular accumulation of superoxide radical (Fig. 3b), there was an increase at 48 h in both groups, most prominent in the group not submitted to splenic artery ligation (p < 0.001). At 120 h this difference between Hx and Hx + Asp groups remained significant (p < 0.05).

**Figure 4:**
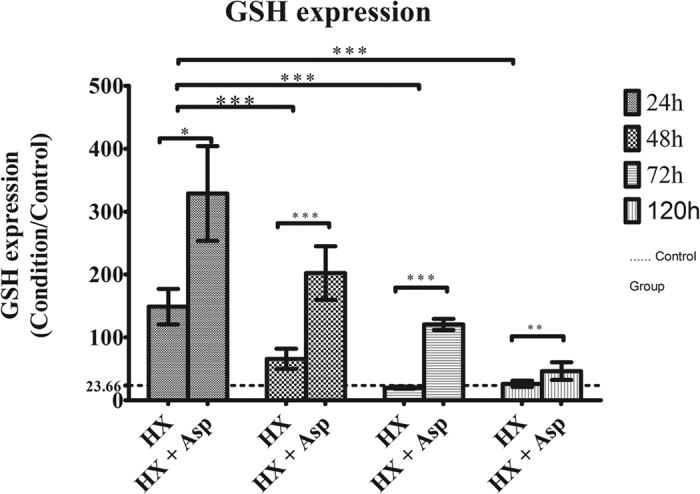
Antioxidant capacity performed by flow cytometry using mercury orange as fluorescent compound. Hepatocytes were extracted at 24, 48, 72 and 120 postoperative hours, from rats submitted to hepatectomy (Hx) or hepatectomy + splenic artery ligation (Hx + Asp). Results are expressed by MFI values of GSH expression ± standard deviation, in hepatocytes extracted from six rats in each group. ***p < 0.001; **p < 0.01; *p < 0.05. — Baseline value in a control group without any procedure.

In order to better characterize the effect of splenic artery ligation after hepatectomy on cellular oxidative stress, it was also measured the reduced glutathione (GSH) anti-oxidant capacity. As shown in Fig. 4, there was an increase of anti-oxidant capacity at 24 h in both groups (p < 0.001), more expressive in the group submitted to splenic artery ligation (p < 0.05). This procedure induced, over time, a decrease of anti-oxidant capacity (p < 0.05).

### Mitochondrial membrane potential estimation

**Figure 5:**
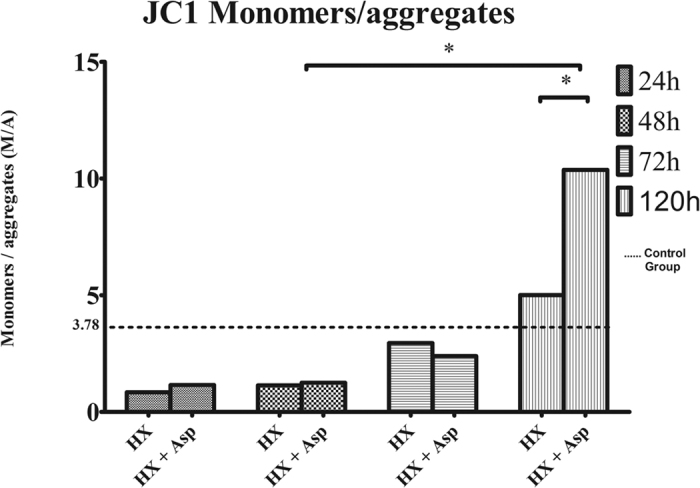
Analysis of mitochondrial membrane potential by flow cytometry using fluorescent probe JC-1. Hepatocytes were extracted at 24, 48, 72 and 120 postoperative hours, from rats submitted to hepatectomy (Hx) or hepatectomy + splenic artery ligation (Hx + Asp). Then, they were isolated and stained with the JC-1 probe. JC-1 probe coexist in monomeric (M) or aggregated (A) form depending on the mitochondrial membrane potential. An increase in the M/A ratio indicates a decrease in the mitochondrial membrane potential. Results are expressed as average of monomers/aggregates ratio ± standard deviation, in hepatocytes extracted from six rats in each group. ***p < 0.001; **p < 0.01; *p < 0.05. — Baseline value in a control group without any procedure.

Mitochondrial signalling regulates the communication between mitochondria and nucleus influencing many cellular responses under normal or pathophysiological conditions^42^. In this context, we analysed the mitochondrial membrane potential by flow cytometry using the fluorescent probe JC-1. As shown in Fig. 5, at 120 h there was a significant decrease of mitochondrial membrane potential in both groups, expressed by monomers-aggregates ratio (M/A) of JC-1 increase, comparing with 48 hours (p < 0.05), being more expressive in the group submitted to splenic artery ligation (p < 0.05).

### Histological analysis - Tissue injury and hepatocyte regeneration

**Figure 6:**
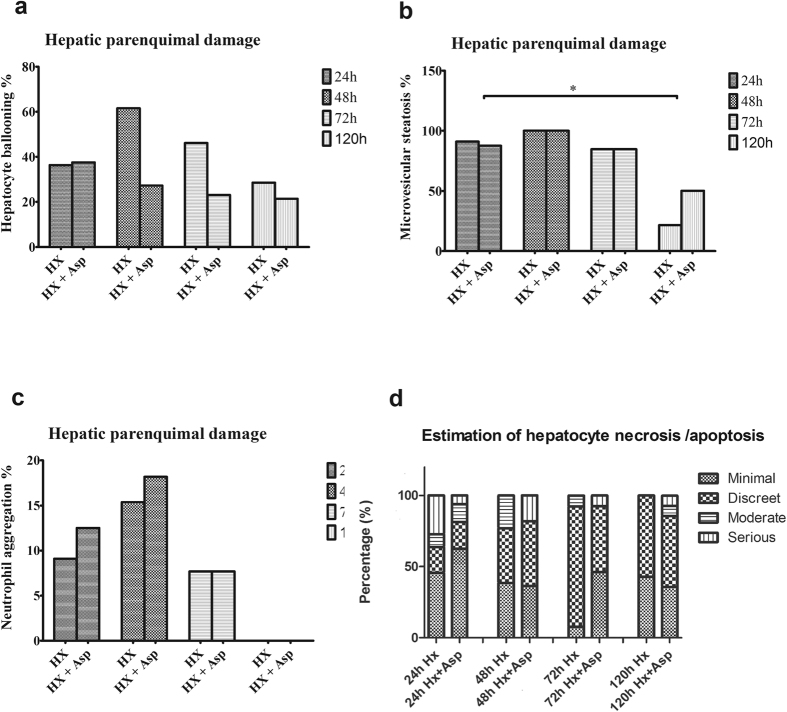
Histological analysis of tissue injury. Sample of liver tissue collected at 24, 48, 72 and 120 postoperative hours, from rats submitted to hepatectomy (Hx) or hepatectomy + splenic artery ligation (Hx + Asp). Results are expressed as median of the percentage for each tissue injury marker: hepatocyte ballooning (**a**), microvesicular steatosis (**b**), neutrophil aggregation (**c**) and estimation of hepatocyte necrosis/apoptosis (**d**). Liver samples were collected from six rats in each group.

**Figure 7:**
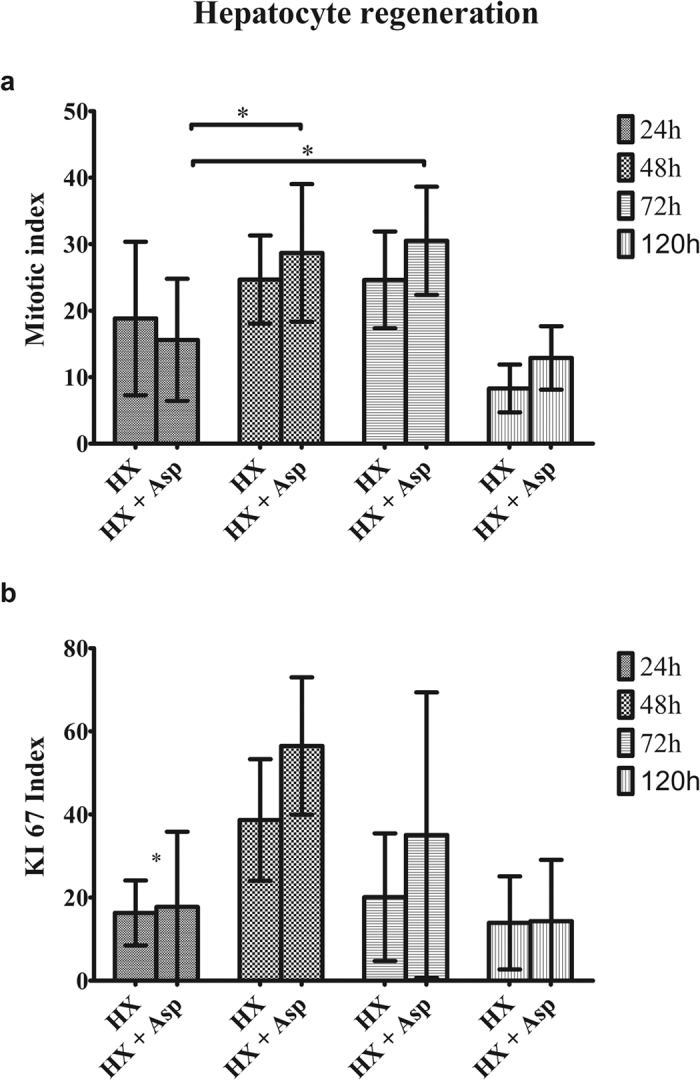
Histological analysis of hepatocyte regeneration. Samples of liver tissue collected at 24, 48, 72 and 120 postoperative hours, from rats submitted to hepatectomy (Hx) or hepatectomy + splenic artery ligation (Hx + Asp). Mitotic activity (**a**) is expressed by average of mitosis counting in 10 consecutive fields of high magnification ± standard deviation, in liver samples collected from at least five rats in each group. Ki-67 (**b**) is expressed by average of percentage of positive hepatocellular nuclei/1000 hepatocytes ± standard deviation, in liver samples collected from six rats in each group. Exception: to observe Ki-67 expression were used 4 rats in each group.

Concerning tissue injury, as shown in Fig. 6, in the Hx group there was an increase of hepatocyte ballooning percentage at 48 h, by comparison with the Hx + Asp group, which became gradually less expressive, but without statistical significance (Fig. 6a). In relation to microvesicular steatosis, there was a decrease in both groupsat 120 h, comparing to previous times (p < 0.05) (Fig. 6b). Regarding the percentage of neutrophil aggregates and the estimated hepatocyte necrosis/apoptosis, there were no statistically differences among groups or over time (Fig. 6c,d). In Fig. 7 we can see the results of hepatocyte regeneration. They show an increase of mitotic index between 48 h and 72 h, more expressive in Hx + Asp groups (p < 0.05) (Fig. 7a). There was a tendency to the increment of Ki-67 expression between 48 h and 72 h, in both groups, most notoriously in Hx + Asp group, although without statistical significance (Fig. 7b). In supplementary material are shown all histological examples.

## Discussion

The results of this study showed that splenic artery ligation produced significant viability increase at 24 h which remained over time, inducing a decrease in intracellular accumulation of peroxides and superoxide radical during the first 48 hours. This surgical approach also allowed antioxidant capacity increment at 24 h, which was reflected in a decrease of half-time normalized liver curve at 48 h and at 72 h and in an increase of mitotic index between the 48 h and 72 h.

Several experimental studies that include pharmacological therapies^18^,^19^,^20^,^21^,^24^ and surgical strategies^25^,^32^,^39^ to modulate portal influx corroborate these findings, demonstrating a mitigating effect on oxidative stress and an increase of hepatocyte viability. Despite splenic artery ligation did not have effects on hepatic extraction fraction and have generated a Tmax increase in the first 24 h, it induced a significant decrease of T_1/2_ at 48 h and 72 h for a significance level exceeding 5% (p = 0.052). This slight increase in hepatic depuration can be explained by the need for a longer period than 5 postoperative days to recover the hepatic function. Our previous research work supports this explanation^5^. Regarding histological analysis of hepatocytes, the splenic artery ligation, when associated with a major hepatectomy, did not entail higher hepatocyte injury. In contrast and although there was no statistical significance, it seems that the modulation of portal inflow tends to protect the remaining liver parenchyma from the injury induced by hepatectomy, which is supported by previous studies which demonstrate attenuation of parenchymal injury following major hepatectomy, through portal inflow modulation^9^,^25^. The mitotic index increased in both groups, between 48 and 72 h, but more significantly in the group where splenic artery ligation was performed (p < 0.05), showing that this surgical manoeuver, by portal inflow modulation, induces a positive effect on cell regeneration, as it has been shown in previous works^39^,^40^. The tendency to increase Ki-67 expression observed between 48 and 72 h, stronger in the Hx + Asp group, corroborates these results. However, the lack of statistical significance, in our opinion, can be due to the small sample in this variable.

The increased viability and regeneration in Hx + Asp group is probably a consequence of oxidative stress reduction at first 48 h and is caused by anti-oxidative capacity increment at the first 24 hours. Despite free radical generation during physiologic and pathologic conditions, cells had an arsenal of cellular defenses including enzymatic defenses, such as superoxide dismutases (SODs), catalase, GSH peroxides, glutaredoxins, and non-enzymatic antioxidants, such as GSH, and vitamins A, C and E. The removal of ROS is efficiently ensured by the coordinated action of these cellular defenses. SODs catalyze the dismutation of superoxide anion into hydrogen peroxide^43^. During the first 24 h posthepatectomy, the SOD activity may be increased which caused the observed increase in intracellular peroxides levels in consequence of superoxide anion dismutation. However, after this time SOD activity may decline, inducing the increase in superoxide anion levels observed at 48 h posthepatectomy and a subsequently decrease in hydrogen peroxide levels. Since we only analysed the intracellular levels of GSH the delay in superoxide formation needs to be further addressed in future studies, namely through SODs activity. Major hepatectomy had been associated with a transitory elevation in GSH levels^44^. Here, we observed that Hx and Hx + Asp induced an increase in GSH levels during the first 48 h after surgery relatively to normal levels (GSH 24 ± 6 MFI; data not shown), deceasing to normal values after 120 h. According to Huang *et al*., cellular GSH increases in regenerating rat liver during the first 24 h or 48 h^44^. This increase in GSH levels may reflect the shifting of cells from G0 to G1 phase or the G1 to S transition^44^, and can explain the higher cell viability, as well as the increased mitotic and Ki-67 indexes, observed in Hx + Asp in comparison to Hx.

Mitochondrial signaling regulates the communication between mitochondria and nucleus influencing many cellular responses under both normal and pathophysiological conditions^42^. During substrate oxidation, mitochondria produce a membrane potential in form of a proton gradient across mitochondrial inner membrane. Several factors can influence mitochondrial membrane potential, including apoptosis, external concentration of growth factors and glucose levels^45^. In the present study, we observed that Hx and Hx + Asp induced an increase in mitochondrial potential (represented by a decrease in monomers/aggregates ratio) in the first 48 h comparing with the group at surgical time (3.78 ± 1.47; data not shown) that decrease at 120 h. In association with the increase in mitotic index and Ki-67 levels, this result may be associated with an increased proliferation. However, 120 h after HX + Asp, it was observed a decrease in mitochondrial membrane potential not associated with cell death. This fact may be due to others factors also associated with a decline in mitochondrial membrane such as a decrease in growth factors levels.

The biomolecular findings evidenced in this paper, in line with previous experimental investigations about portal inflow modulation^9^,^18^,^19^,^20^,^21^,^24^,^25^,^29^,^32^,^39^,^40^, support the improved survival and decreased morbidity observed in previous clinical studies researching portal inflow modulation^33^,^38^.

In fact, portal inflow modulation may change future hepatic resection surgery and liver transplantation^2^,^5^,^9^. Nowadays, it could allow operate patients whose hepatic lesion and dysfunction, conditioned by their previous pathology or by neoadjuvant therapy, currently preclude them from major liver resections. In addition, it could allow the use of smaller liver grafts, without increased risk for SFSS, which determines lesser iatrogeny for the donor and makes possible the increase of compatible donor’s pool. Besides, it may also increase resection rate in metastatic disease, by allowing lower FLR minimum needed to avoid PHLF. Perform portal inflow modulation, through the splenic artery ligation, allows minimize the deleterious effects of portal venous overpressure and prevent the SFSS, using a reproducible procedure, without compromising hepatocellular function, as we demonstrate in this work. It also opens doors to, in a future clinical approach, evaluate its effects on morbidity and survival of patients undergoing MH or LDLT.

In summary, this study shows that the splenic artery ligation combined to 85% hepatectomy in a murine model, allows portal inflow modulation, promoting an increase in hepatocellular viability and regeneration, without impairing hepatic function, probably by inducing a less marked elevation of oxidative stress at first 48 hours after major hepatectomy.

## Material and Methods

All methods and experiments were carried out in accordance with Coimbra University guidelines. Animals were housed and kept in accordance with the Institution of Animal Care of the University of Coimbra. All experiments protocols were carried out after approval by Faculty of Medicine of Coimbra Ethical Committee (ProcNumb 116-2011).

### Animals

Eight-weeks-old male Wistar rats weighing between 200 and 300 g, were used. Animals were fed a laboratory diet with water and food *ad libitum* until surgery and were kept under constant environment conditions with 12-hour light-dark cycle.

Forty-eight rats divided into two groups underwent 85% hepatectomy (Hx group n = 24) or 85% hepatectomy plus splenic artery ligation (Hx + Asp group, n = 24). Each group was divided into four sub-groups according to time of postoperative evaluation set at 24 (n = 6), 48 (n = 6), 72 (n = 6) and 120 (n = 6) postoperative hours. A control group of 10 animals were sacrificed on the 7^th^ postoperative day.

### Operative procedures

All animals were anesthetized with ethoxyethane by inhalation. Abdomen was opened through a midline incision and liver was freed of its ligaments. 85% hepatectomy with removal of the left lateral and median lobes were performed in all animals. Splenic artery ligation was performed on a control group (50%). Under our surgical conditions, a few branches of splenic artery were intact^5^,^40^,^46^,^47^. In each time after surgery (24, 48, 72 and 120 h) several variables were evaluated, using different techniques such as: flow cytometry to assess cell viability, oxidative stress levels, antioxidant capacity and mitochondria membrane potential; histopathologic analysis to evaluate hepatic tissue damage and histological regeneration (mitotic index and Ki-67) and nuclear medicine evaluation in order to estimate hepatic extraction function.

### Hepatic extraction fraction (HEF) evaluation by radioisotopic methods

For the HEF calculation, an IDA derivative, 3-bromo-2,4,6-trimethylacetanilidoiminodiacetic acid labelled with technetium-99 m (99 mTc-mebofrenin) was used. The labelling procedure was performed as recommended by the manufacturer^5^,^13^.

With the total counts from the ROIs drawn over heart and liver, time-activity curves were generated and HEF calculated by deconvolution analysis. Additionally, the Tmax and half-time of normalized liver curve (T_1/2_) were calculated, according to data processing described by Tralhão *et al*.^13^.

### Liver cell isolation

Liver cells were isolated from liver fragments collected after animal sacrifice by using collagenase (Collagenase-Hepatocyte-Qualified – Gibco/BRL) as described elsewhere^48^. After liver cell isolation, viability and death, oxidative stress and mitochondrial membrane potential were evaluated by flow cytometry. Flow cytometry analysis was performed using a six-parameter, four-color FACS Calibur^TM^ flow cytometer (Becton Dickinson) equipped with a 15 mW argon laser. For each assay 10^6^ cells were used and at least 10^4^ events were collected by acquisition using Cell Quest software (Becton Dickinson) and analysed using Paint-a-gate software (Becton Dickinson)^13^,^49^,^50^,^51^.

### Cell death and viability analysis

Isolated hepatocytes were stained simultaneously with Annexin-V (AV), labelled with the fluorescent probe, fluorescein isothyocianate (FITC), and propidium iodide (PI), according to manufacturer’s recommended protocol (Immunotech Kit). This assay discriminates among intact (AV−/PI−), early apoptotic (AV+/PI−), late apoptotic (AV+/PI+) and necrotic cells (AV−/PI+). The results are shown as percentage of each cell type, as it was previously described by Mamede *et al*.^13^,^51^.

### Oxidative Stress

The ROS accumulation in hepatocytes, namely peroxides, was evaluated using 2′,7′-dichlorodihydrofluorescein diacetate (DCFH2-DA) (Invitrogen^TM^), a stable no fluorescent lipid permeable compound which is converted into DCFH2 by intracellular esterases. Then, DCFH2 is oxidized by intracellular ROS to form the impermeable fluorescent compound DCF that emits green fluorescence, after light excitation of 488 nm, proportionally to intracellular ROS levels^13^,^51^,^52^.

Preparation protocol for DCF detection in hepatocytes was performed as previously described. The results are shown as mean fluorescence intensity (MFI) values^13^,^50^.

Assessment of intracellular superoxide radical production was performed by flow cytometry using dihydroethidium (DHE). This compound readily crosses cellular membranes and is converted by the superoxide radical into ethidium, a red fluorescent compound that merges DNA and remaining inside the cell. This reaction is relatively specific for superoxide radical with minimal oxidation by hydrogen peroxide, hypochlorous acid or nitrite. Ethidium detection protocol was performed with an excitation wavelength of 620 nm, according to described by Mamede *et al*. Results are shown as MFI values^51^,^52^.

Reduced glutathione (GSH) is the major endogenous antioxidant, directly participating in neutralization of free radicals and reactive oxygen species. Its intracellular concentration is proportional to the estimated antioxidant capacity of the cell. The GSH expression was performed by flow cytometry using as fluorescent compound the mercury orange, with an excitation wavelength of 620 nm, according to Mamede *et al*. Results are shown as MFI values^51^.

### Mitochondrial membrane potential measurement

The integrity of the inner mitochondrial membrane can be evaluated measuring the potential gradient across this membrane, using the dye 5,5′,6,6′-tethrachloro-1,1′,3,3′-tethraethylbenzimidazolcarbocyanine iodide (JC-1). JC-1 is a molecule able to selectively enter the cell and exists in 2 forms, aggregates (A) and monomers (M), depending on the polarization/depolarization state of mitochondrial membrane. When the mitochondrial membrane potential is high, JC-1 forms aggregates that emit red fluorescence (590 nm). In turn, if the mitochondrial membrane potential decreases or the membrane is depolarized, JC-1 is excluded from mitochondria and remains in the cytoplasm in the form of monomers that emit green fluorescence (529 nm). The ratio between the intensities of green and red fluorescence (M/A), determined by flow cytometry, provides a mitochondrial membrane potential estimation^51^. The results are performed by flow cytometry, as previously described^13^,^50^,^51^ and are shown as monomer/aggregate ratio.

### Histopathological analysis

For each collected liver fragment, macroscopic description was made according to size, external surface and cutting features as colour and consistency. Then the fragments were automatically processed (Shandon Excelsion ES^®^) and included in paraffin. For microscopic examination, samples were cut at 3 μm thickness, processed and stained by haematoxylin and eosin^53^.

The stained histological sections were evaluated for the presence or absence of tissue damage, hepatocyte ballooning, microvesicular steatosis and polymorphonuclear neutrophils aggregates^54^,^55^.

Hepatocyte necrosis/apoptosis was estimated based on the number of necrotic foci per field using medium magnification (10x objective) and classified in four categories: minimal (<2 foci); discrete (2–4 foci); moderate (5–10 foci) and severe (>10 foci or “bridging”)^56^,^57^.

The mitotic activity was determined by the number of mitosis counted in ten consecutive fields using high power magnification (objective 40x/0.57)^58^.

For the immunohistochemical procedure, the macroscopic recording and processing of the samples were performed within 48 hours after collection, using Novocastra Novolink detection kit (Leica^®^) according to a protocol described by Tsutsumi *et al*.^59^.

The immunohistochemical study with Ki-67 antibody allowed a semi-quantitative evaluation of proliferative index by determining the percentage of positive hepatocellular nuclei in 1000 hepatocytes^60^.

### Statistical analysis

Statistical analysis was performed using the IBM SPSS^®^ statistical software (version 21.0). Shapiro–Wilk test was used to evaluate normal distributions for continuous variables. For variables with normal distribution parametric tests were used, otherwise the tests used were not parametric. To compare differences between Hx and Hx + Asp groups, the Mann-Whitney U-test or the independent samples t-test was used. We performed analysis of variance or Kruskal–Wallis test to compare continuous variable time among four groups (24, 48, 72 and 120 h). Multiple comparisons were performed considering the Bonferroni correction.

All significance tests were two-tailed and p < 0.05 was considered significant.

## Additional Information

**How to cite this article**: Carrapita, J. *et al*. Impact of splenic artery ligation after major hepatectomy on liver function, regeneration and viability. *Sci. Rep.* **6**, 34731; doi: 10.1038/srep34731 (2016).

## Supplementary Material

Supplementary Information
