# Supplementary material for: Impact of splenic artery ligation after major hepatectomy on liver function, regeneration and viability
Source: Sci Rep. 2016 Oct 11;6:34731. doi: 10.1038/srep34731 (PMC5057079; doi:10.1038/srep34731)

Supplementary material

Title of manuscript: Impact of splenic artery ligation after major hepatectomy on liver function, regeneration and viability

Authors:

Jorge Carrapita*[1,2], Ana Margarida Abrantes[3,4,5], Sofia Campelos[6], Ana Cristina Gonçalves[4,5,7], Dulce Cardoso[8], Ana Bela Sarmento-Ribeiro[4,5,7,9], Clara Rocha[10,11], Jorge Nunes Santos[2], Maria Filomena Botelho[3,4,5], José Guilherme Tralhão[3,4,5,12], Olivier Farges[13], Jorge Maciel Barbosa[1,14]

[1] General Surgery Department of Vila Nova de Gaia/Espinho Hospital, Portugal
[2] Institute of Biomedical Sciences Abel Salazar, University of Oporto, Portugal
[3] Biophysics Unit, Faculty of Medicine, University of Coimbra, Portugal
[4] CNC.IBILI, Faculty of Medicine, University of Coimbra, Portugal
[5] Centre of Investigation on Environment, Genetics and Oncobiology (CIMAGO), Portugal
[6] Pathologic Anatomy Department of Vila Nova de Gaia/Espinho Hospital, Portugal
[7] Laboratory of Oncobiology and Hematology (LOH), University Clinic of Hematology and Appied Molecular Biology Unit, Faculty of Medicine, University of Coimbra, Portugal
[8] Nuclear Medicine Department, University Hospital of Coimbra, Portugal
[9] Clinical Hematology Department, Coimbra University Hospital Centre (CHUC), Portugal
[10] ESTESC-Coimbra Health School Department Complementary Sciences, Polytechnic Institute of Coimbra, Portugal.
[11] Institute for Systems Engineering and Computers at Coimbra (INESCC), Portugal
[12] Surgery A, Surgery Department of Coimbra University Hospital, Faculty of Medicine, University of Coimbra, Portugal

Fig. 8. Hepatocyte ballooning, in each group, at 48h. (a) Hx group. (b) Hx+Asp group.

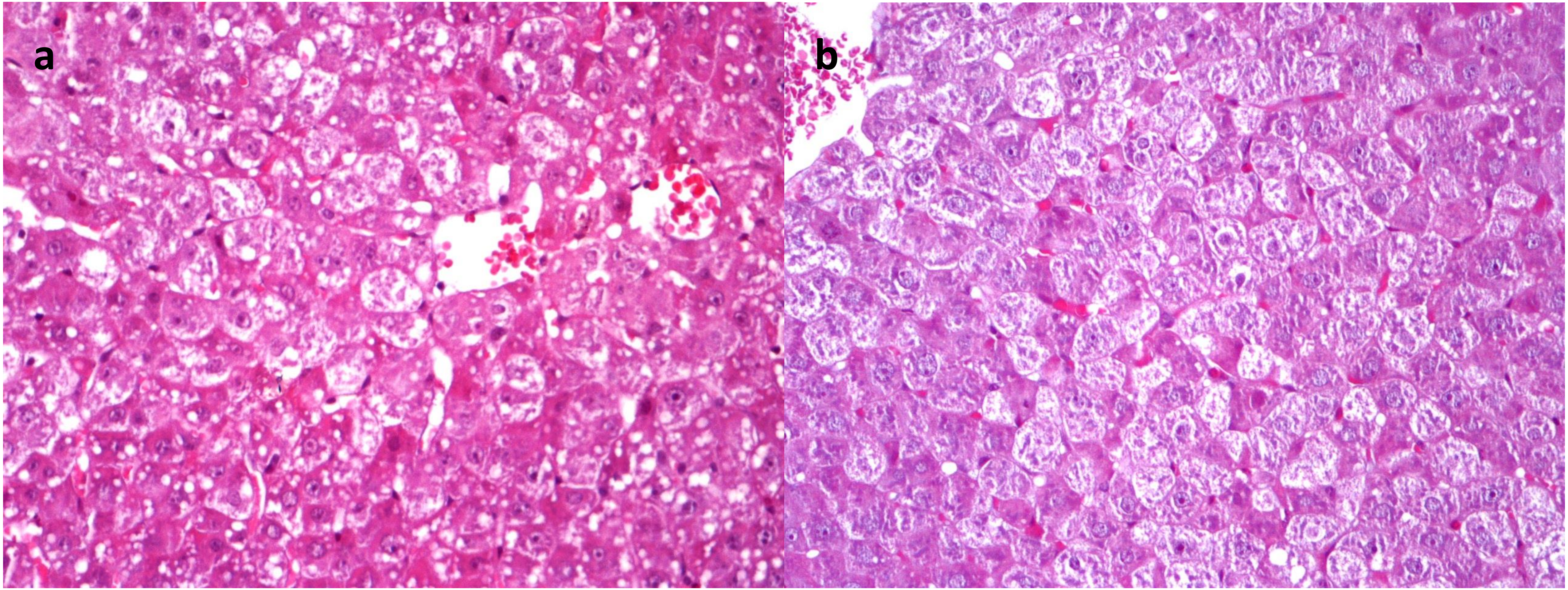

Fig. 9. Decrease of microvesicular steatosis over time. **(a)** Hx+Asp group at 24h. **(b)** Hx+Asp group at 120h.

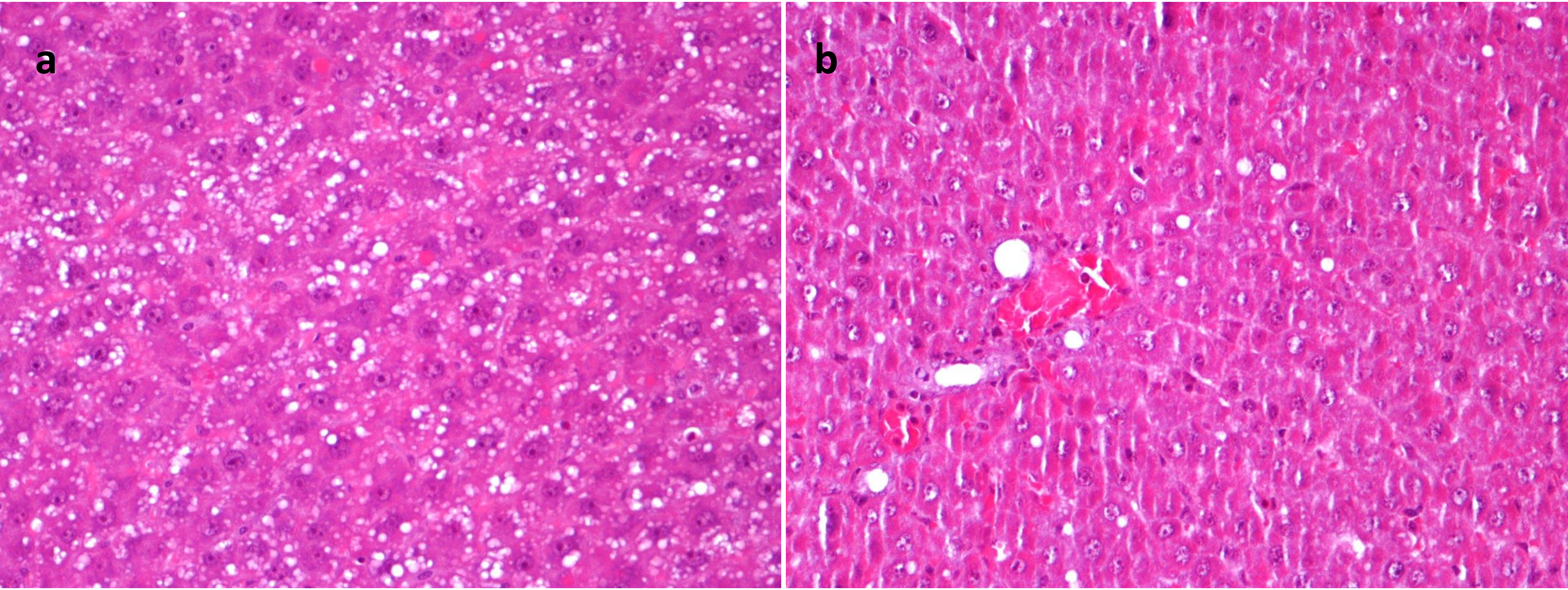

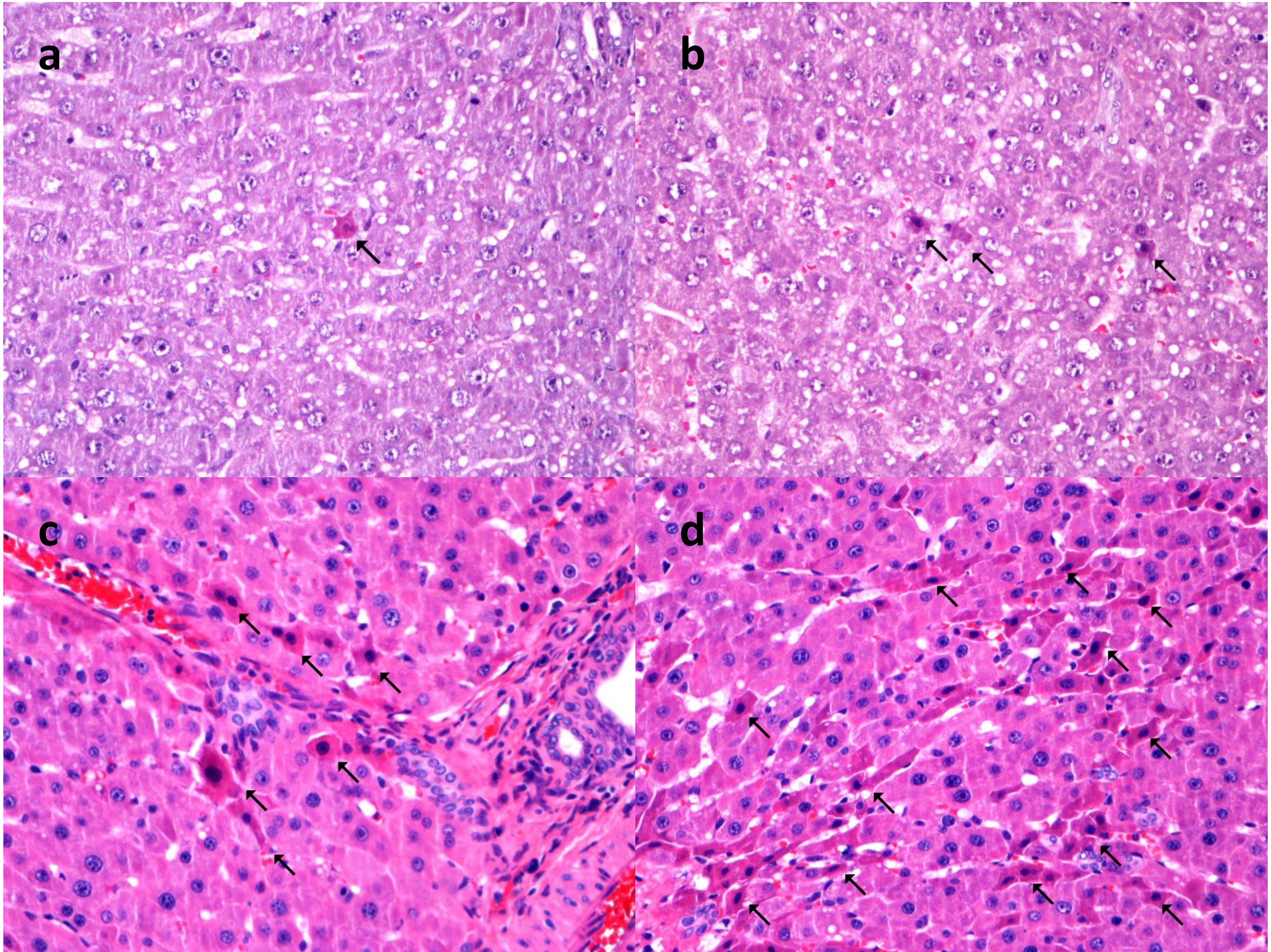

Fig. 10. Necroinflammatory activity score (based on the Modified Histology Activity Index).
(a) Minimal (<2 foci).
(b) Discrete (2-4 foci).
(c) Moderate (5-10 foci). **(d)** Severe (>10 foci or "bridging”).
↗ Focal lytic necrosis and apoptosis.

Fig. 11. (a) High mitotic index at 72h, on the group HX+Asp. **(b)** Staining about a half of hepatocyte nuclei with Ki67 at 48h, on the HX+Asp group.

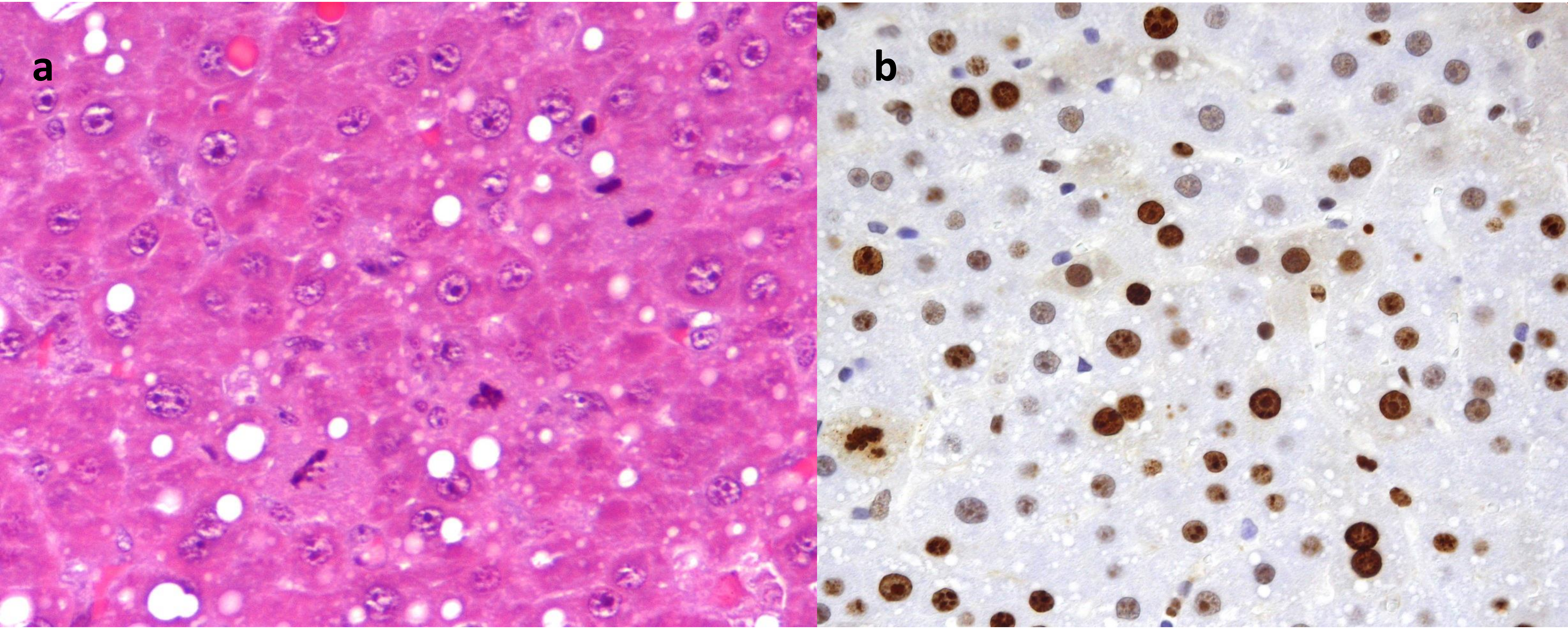

Supplement: Supplementary Information [file srep34731-s1.pdf]
